# Representations of protein structure for exploring the conformational space: A speed–accuracy trade-off

**DOI:** 10.1016/j.csbj.2021.04.049

**Published:** 2021-04-28

**Authors:** Guillaume Postic, Nathalie Janel, Gautier Moroy

**Affiliations:** a Université de Paris, BFA, UMR 8251, CNRS, ERL U1133, Inserm, F-75013 Paris, France; b Université de Paris, BFA, UMR 8251, CNRS, F-75013 Paris, France

**Keywords:** Protein structure prediction, Statistical potentials, Coarse-grained models, Protein folding, Low-resolution representation

## Abstract

The recent breakthrough in the field of protein structure prediction shows the relevance of using knowledge-based based scoring functions in combination with a low-resolution 3D representation of protein macromolecules. The choice of not using all atoms is barely supported by any data in the literature, and is mostly motivated by empirical and practical reasons, such as the computational cost of assessing the numerous folds of the protein conformational space. Here, we present a comprehensive study, carried on a large and balanced benchmark of predicted protein structures, to see how different types of structural representations rank in either accuracy or calculation speed, and which ones offer the best compromise between these two criteria. We tested ten representations, including low-resolution, high-resolution, and coarse-grained approaches. We also investigated the generalization of the findings to other formalisms than the widely-used “potential of mean force” (PMF) method. Thus, we observed that representing protein structures by their β carbons—combined or not with Cα—provides the best speed**–**accuracy trade-off, when using a “total information gain” scoring function. For statistical PMFs, using MARTINI backbone and side-chains beads is the best option. Finally, we also demonstrated the necessity of training the reference state on all atom types, and of including the Cα atoms of glycine residues, in a Cβ-based representation.

## Highlights

• We compare ten structural representations, either atomistic or coarse-grained.
• Thus, ten distance-dependent statistical potentials of mean force (PMF) were built.
• The Cβ-only and Cα + Cβ representations provide the best speed–accuracy trade-off.
• Including glycines through Cα, in a Cβ-only representation, yields a higher accuracy.
• We generalize the conclusions to the total information gain (TIG) scoring function.

## Graphical abstract

**Figure:**
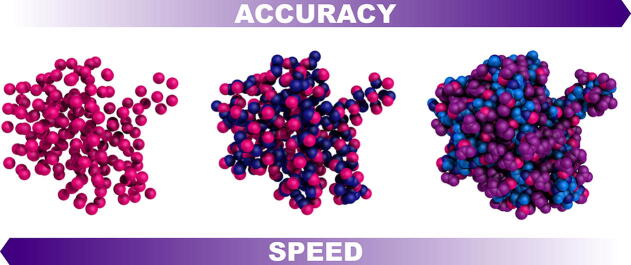

## 1 Introduction

The protein folding ranks among the most important unsolved problems in science [1]. For fifty years, since the Nobel Prize-winning work of C. B. Anfinsen for demonstrating the thermodynamic spontaneity of this process, researchers have wondered how to predict the three-dimensional conformation of the polypeptide chain, based on the sole amino acid sequence. This scientific question could even be dated ten years earlier, since the X-ray crystallographic study of the structure of myoglobin, by M. F. Perutz and J. C. Kendrew, also awarded with a Nobel Prize in Chemistry. The critical nature of the problem arises from the facts that (i) protein function results from the 3D structure, through dynamical features and interactions with other biomolecules, and (ii) the experimental determination of native conformations remains challenging, despite the recent advances in cryogenic electron microscopy techniques. Therefore, the development of a computational method that could accurately predict protein fold would have a profound impact across all areas of biology, from fundamental to applied research.

The prediction of protein structure from sequence requires *sampling* the conformational space, using an algorithm guided by a *scoring* function. Exploring all possible conformations is not computationally feasible, as their number would be ~10^30^ for an average size protein of 100 residues [2]. Thus, different sampling approaches have been employed [3]: optimization and heuristic algorithms, in *ab initio* modeling; the detection of experimental structures used as templates compatible with the sequence, in comparative modeling; and the assembly of structural fragments in *de novo* modeling. For all methods, the objective is to find a structure that minimizes a cost function. The latter is designed to approximate Gibbs free energy, which is supposed to be minimal for the native conformation—by Anfinsen’s hypothesis [4]. The impossibility of an exhaustive conformational sampling prevents any rigorous calculation of the entropy change of the system (protein chain and solvent) during the folding reaction. As a result, physical energy functions rarely meet success, most likely because they account for the entropic contribution to the protein folding (e.g. [5], [6]) in a way that is either too approximate or computationally inefficient. Statistical potentials, as opposed to physical potentials, are scoring functions obtained from statistics on native (*i.e.* experimental) protein structures—thus also referred to as knowledge-based potentials. Initially devised by R. L. Jernigan and S. Miyazawa [7], this type of scoring functions was later developed by M. J. Sippl, with the now widely-used “potential of mean force” (PMF) method [8]. Being irrelevant to Boltzmannian statistical mechanics, this method should be understood as a consequence of Bayesian probability theory [9], [10], [11], [12]. Statistical PMFs have continuously proven useful in numerous applications, which all boil down to predicting the lowest-energy conformations: protein folding [13], molecular docking [14], protein stability [15], [16] or, most recently, protein solubility and aggregation [17], [18]. Thus, over the past three decades, statistical PMFs have been successfully used, be it directly or included among the terms of so-called “physics-based” energy functions, such as that of the fragment-based prediction method Rosetta [19].

With the advent of machine learning techniques, composite scoring functions have been created by combining statistical PMFs based on different structural features (e.g. interatomic distances, dihedral angle values, or solvent accessibility), the weight of each component being determined by methods such as support-vector machines [20], [21]. Artificial neural networks have also been used to optimize single-feature statistical PMFs [22]. Most recently, researchers have proposed AlphaFold, a deep learning-based method capable of predicting protein structure from sequence more accurately than do the research groups competing in the Critical Assessment of Structure Prediction (CASP) experiment [23]. The scoring function of AlphaFold is a statistical PMF—which depends on interatomic distances and torsion angles—built by training a convolutional neural network on both native protein structures and multiple sequence alignments (MSAs). In this way, the generated the scoring function is improved by incorporating evolutionary information.

A key factor in predicting protein folding is the structural representation of the problem. Interestingly, AlphaFold uses a low-resolution one, by only considering β-carbon atoms of the protein molecule. This could be justified by the need for a scoring function that would be fast enough for a sufficient sampling of the conformational space, while maintaining high accuracy. Nevertheless, this choice is not explained within the article, nor connected to any published reference. It appears that D. T. Jones, one of the AlphaFold authors, has previously published a Cβ-only statistical PMFs used for positioning transmembrane domains within the lipid bilayer [24]. Still, such examples are too scarce and specific for accepting the advantage of this representation as common knowledge. As far as we know, there is no work in the literature that compares the performance of low- and high-resolutions in building statistical scoring functions. In this report, we address this question of the structural representations for exploring the configuration space of protein chains, by generating multiple distance-dependent statistical PMFs and studying their differences in terms of speed and accuracy.

## 2 Methods

### 2.1 Representations of protein structure

A total of 10 structural representations, including both low- and high-resolutions, were tested (Table 1). We also tested coarse-grained models, as defined for the MARTINI force field [25], [26]. The latter represents protein structures with backbone beads (BB) and side-chain beads (SC), the number of which varies depending on the residue type.

**Table 1:** The selected representations of protein structure, categorized according to their resolution. The third column presents the number of atom pairs counted for all the native structures of the training dataset.

Category	Atom type	Total pairs
Low-resolution	Cα	7,467,788
	Cβ	7,204,580
	Cα + Cβ	27,421,952
	backbone	119,826,211
	backbone + Cβ	180,909,149
High-resolution	all-atom	440,201,439
	side-chains	104,858,474
Coarse-grained	BB	7,545,600
	SC	12,820,176
	BB + SC	35,001,152

**Fig. 1:**
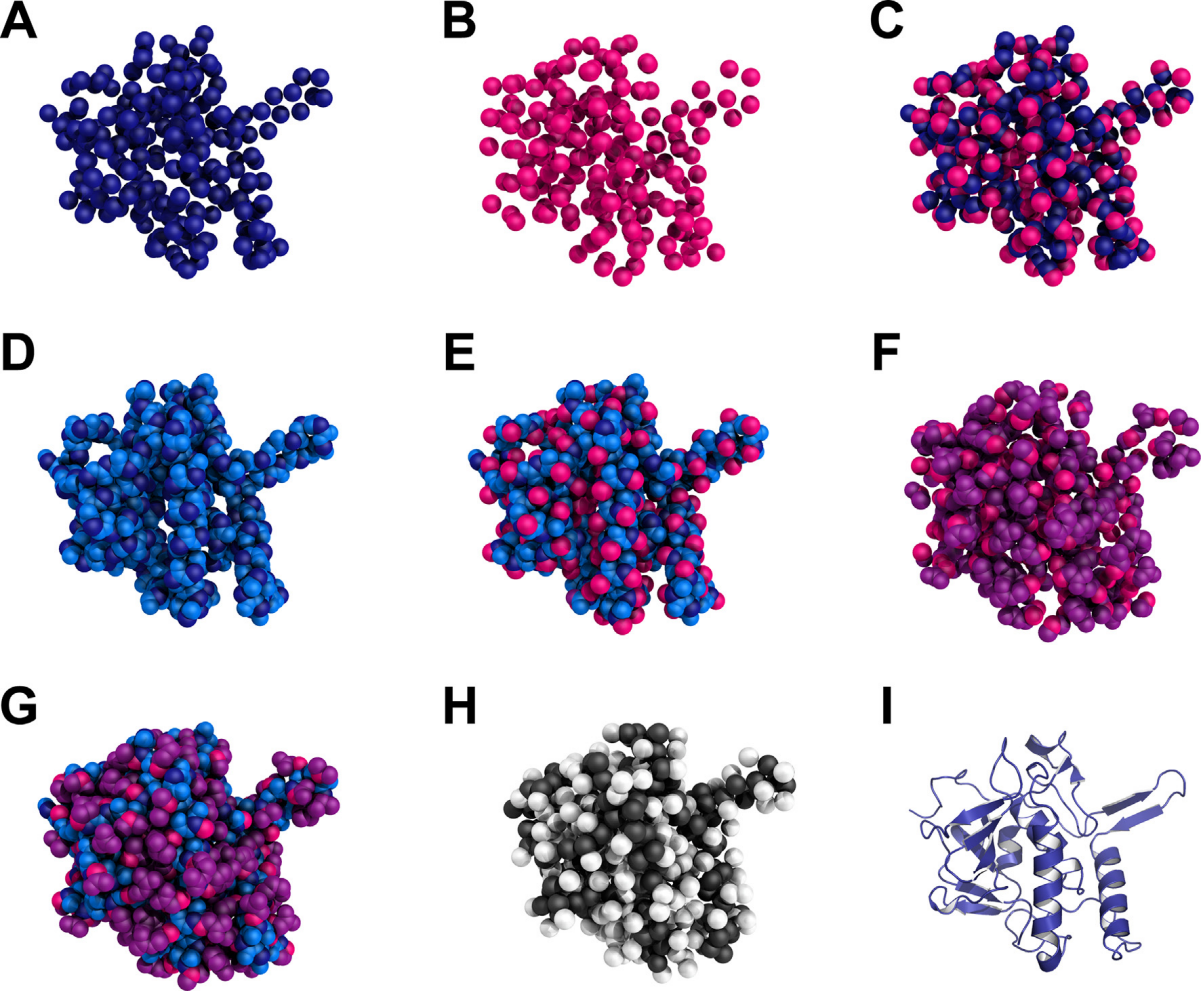
Structure of the peptidoglycan hydrolase RipA, from *Mycobacterium tuberculosis* H37Rv (PDB code: 3ne0), in increasing-resolution order: (A) Cα, (B) Cβ, (C) Cα + Cβ, (D) backbone, (E) backbone + Cβ, (F) side-chains, (G) all-atom. The MARTINI coarse-grained model (H) is represented with backbone beads in black and side-chain beads in white. (I) Cartoon representation of the protein.

For the sake of completeness, we included an unusual “side-chain-only” representation, so that the information content of the side-chain atoms can be assessed in a direct manner, rather than by deduction—*i.e.* by comparing the all-atom- and backbone-based results. Fig. 1 illustrates the different representations, with a protein from the testing set described below. For the Cβ-only representation, the glycine residues were represented by their Cα. Therefore, the difference between the Cα and Cβ representations regarding the number of atom pairs may stem from missing Cβ atoms and/or a threshold effect: for a “XXX” residue, the “XXX-Cα to GLY-Cα” distance may be below the upper limit, while the “XXX-Cβ to GLY-Cα” distance may exceed the threshold and, thus, be discarded. A Cβ-only representation that excludes glycines has also been used in this study and its number of atom pairs was 6,275,363.

### 2.2 Scoring functions: Functional forms

To evaluate the different structural representations, we built interatomic statistical potentials following a Bayesian formulation of Sippl’s PMF, similar to that described in AlphaFold [23]—minus the deep learning on MSAs. Thus, two distance distributions were derived from a set of native structures: one is conditioned on the residue types of the atom pair (conditional model, *M*_1_), whereas the other is not (background model, *M*_2_, the so-called “reference state”). The score of a protein conformation is then calculated as the negative log-likelihood ratio of the distances under the statistical models *M*_1_ and *M*_2_, summed over all pairs of atoms *i*, *j*:

(1) $$\mathrm{score}=-\sum_{i,j,i\neq j}\text{log}\left(\frac{P\left(d_{\mathrm{ij}}|M_{\text{1}}\right)}{P\left(d_{\mathrm{ij}}|M_{\text{2}}\right)}\right)$$

where *P*(*d*_ij_|*M*_1_) and *P*(*d*_ij_|*M*_2_) are the observed probabilities for two atoms *i* and *j* to be separated by a distance *d*, with and without consideration of the atom types, respectively. Often improperly labeled “energy”, the score actually measures the relative support of the statistical models *M*_1_ and *M*_2_, by the distances observed in the protein conformation evaluated. Thus, the more negative the score, the more the assessed structure supports a native distribution of interatomic distances (*M_1_*), over a random one (*M_2_*). This means that the score does not estimates the free energy of the protein fold, but it can be roughly interpreted as such (*i.e.* the lower the better). Rather than distance histograms, we used kernel density estimations to compute these frequencies. The bandwidths of the Gaussian kernels were selected using Scott’s rule-of-thumb [27]. The distances were then discretized into bins of 0.5 Å. Residues separated by less than 3 positions in the sequence were not processed, and distance cutoffs of 17.0 Å and 15.0 Å were applied for the training and scoring procedures, respectively.

Recently, we have challenged the statistical and physical validity of such logarithm-based scoring functions [28]. In this previous work, we presented a new method named “total information gain” (TIG) and demonstrated both its theoretical and practical advantage over the statistical PMFs. Here, to verify whether our conclusions about the different representations can generalize to alternative equations, the benchmark also includes the TIG score, which simply consists of replacing the log-likelihood ratio in Eq. (1) by a relative difference calculated as:

(2) $$\frac{P\left(d_{\mathrm{ij}}|M_{\text{1}}\right)-P\left(d_{\mathrm{ij}}|M_{\text{2}}\right)}{P\left(d_{\mathrm{ij}}|M_{\text{2}}\right)}=\frac{P\left(d_{\mathrm{ij}}|M_{\text{1}}\right)}{P\left(d_{\mathrm{ij}}|M_{\text{2}}\right)}-\text{1}$$

This scoring function was implemented by modifying the MyPMFs algorithm [29] and by using the same parameters and dataset as for the statistical PMFs.

### 2.3 Training and testing datasets

The scoring functions have been trained on a non-redundant (sequence identity ≤ 20%) set of 1917 protein chains, which was selected for our previous work [28]. All the native structures were determined by X-ray crystallography, with a resolution ≤ 1.6 Å and a R-factor ≤ 0.25. Importantly, this dataset was filtered by using PISCES [30], [31] (also with a 20% identity cutoff), to ensure independence from the testing dataset. The latter is the 3DRobot benchmark [32], which contains 300 models for 200 single-domain proteins. With the native conformations, this represents a total of 60,200 structures. This dataset has been specifically designed for benchmarking purposes and ensures a balanced evaluation of the performance, as (i) it is made of non-homologous proteins, and (ii) each of the 200 native structures has been uniformly altered to generate 300 decoys [32]. Four subsets of the 3DRobot dataset have been defined, based on the structural similarity with the native conformation, as measured by the TM-score [33]. Thus, ‘‘near-native”, ‘‘good”, ‘‘medium”, and ‘‘poor” quality models are defined by three TM-score thresholds at 0.8, 0.6, 0.4, respectively. Finally, the protein structures of both the training and testing datasets were converted into MARTINI coarse-grained models, by using the Python script “martinize.py” (version 2.6) [34]. It ran successfully for the all the decoys of 3DRobot. For some native structures, however, the program threw an error due to missing atoms in the PDB file. This was corrected by using re-refined and rebuilt structures from the PDB-REDO database (https://pdb-redo.eu/) [35], [36].

### 2.4 Performance assessment

The performance of each structural representation was assessed by the capacity of the corresponding scoring function to rank protein models according to their true quality (“ground truth”), the latter being measured by the TM-score to the native conformation. Thus, three evaluation procedures have been implemented. For the first one, all pairs of models from the 3DRobot set were ranked by each scoring function, and the accuracy was calculated as the proportion of correct rankings. As trying to distinguish between very similar models would be meaningless, a minimum difference in terms of similarity to the native structure was set to 0.1 TM-score. For the second evaluation procedure, the correlation between the predicted and the true model qualities—*i.e.* the correlation between the scoring function and the TM-score—was measured for each set of 300 decoys from 3DRobot. The Pearson, Spearman’s rank, and Kendall’s rank correlation coefficients were computed and averaged over the 200 proteins. It should be noted that this second evaluation allows comparison with results obtained independently for other state-of-the-art model quality assessment programs [21]. Finally, the third procedure concerns the average ranking (as predicted by the scoring function) of structures belonging to the ‘‘near-native” and ‘‘good” models, as described above. For these two categories, the higher the rank, the better. This test was also applied to the ‘‘poor” models, for which the lower the rank, the better. The statistical significance of the observed differences between accuracies was determined by com- paring the distributions of correct and wrong rankings, using the Wilcoxon signed-rank test, with an α error of 0.05.

Along with accuracy, speed is the other performance criterion for selecting a representation of protein structure. Therefore, for each scoring functions, the time taken to score the 60,200 models from 3DRobot was measured. The computations were performed on a personal computer with an Intel® Xeon™ Silver 4116 CPU at 2.10 GHz and 16896-KB cache size, running the Linux Ubuntu 20.04 LTS operating system. The Bash command “time” was used to measure the CPU time used, which is the sum of the “sys” and “user” output values.

## 3 Results and discussion

### 3.1 Accuracy benchmark

When devising a scoring function to evaluate the folds found by sampling protein conformational space, accuracy is the primary criterion. Here, we have compared the accuracy of statistical potentials based on 10 different representations of protein structure, and following 2 different formalisms (Table 2). For the near-native and good models, the statistical PMF performed the worst with the Cα. The use of Cβ resulted in a dramatic improvement (near-native = +15.1%; good = +5.5%) and the Cα + Cβ further increased the accuracy significantly. Using the four backbone atoms (N, Cα, C and O) placed the scoring function in between the Cα- and Cβ-based ones. For the near-native category, the addition of Cβ to the backbone representation did not outperform the Cβ-only one, but it did for the good models. For both categories, the exclusive use of side-chains ranked second among the seven atomic representations, while the all-atom one ranked first—with 88.0% and 85.8% of correct rankings, for the near-native and good categories, respectively. The MARTINI backbone beads “BB”, which are approximately as numerous as the Cα and Cβ atoms (Table 1), performed only slightly better than the Cα. However, the side-chains beads “SC” equaled the Cβ-only representation. Finally, the statistical PMF using of all MARTINI beads ranked second out of ten, only topped by the all-atom representation.

**Table 2:** Accuracy in ranking models pairwise (n = 60,200) of the different protein structure representations. A random ranking would yield a 50.0% accuracy. A model quality labeled ‘‘near-native”, ‘‘good”, ‘‘medium”, or ‘‘poor” corresponds to TM-score intervals [1.0, 0.8[, [0.8, 0.6[, [0.6, 0.4[, or [0.4, 0.0], respectively.

		Accuracy (%)									
Model quality	Scoring	Cα	Cβ	Cα + Cβ	backbone	backbone + Cβ	side-chains	all-atom	BB	SC	BB + SC
Near-native	PMF	67.8	82.9	84.7	70.9	79.4	85.7	88.0	70.8	82.7	86.5
Good		70.6	76.1	80.6	75.5	80.1	82.5	85.8	71.4	77.5	83.5
Medium		71.2	67.8	74.5	71.9	74.6	75.1	78.2	59.3	72.0	75.1
Poor		68.4	66.4	70.0	68.8	70.6	69.3	72.1	65.2	65.8	69.1
Near-native	TIG	71.6	85.2	86.5	55.7	64.8	59.5	63.1	54.3	62.2	66.1
Good		75.1	80.8	83.3	62.5	68.8	61.5	66.0	69.0	67.1	72.5
Medium		75.2	74.0	77.8	65.3	69.4	58.3	64.5	65.6	64.7	69.9
Poor		70.6	71.6	72.5	63.6	67.4	60.9	64.9	67.4	63.7	67.2
											

By looking at the four model qualities, three observations can be made about statistical PMFs: (1) the accuracy decreases with the model quality; (2) the accuracy increases with the number of atoms and beads in the structural representation; (3) observations 1 and 2 do not apply to the Cα, backbone, backbone + Cβ, and backbone beads representations. The first observation is simply due to the training on native protein conformations, which made the resulting statistical potentials best adapted to near-native predictions. Assessing poor models would have required interatomic distance distributions derived from protein structures of the proper quality (see also Section 2.2). The second observation is also intuitive, as it shows that using a larger amount of informative data makes the quality assessment more accurate. The third observation is related to the strong correlation between the spatial coordinates of backone atoms. Thus, their interatomic distances carry more redundancy than information, which deteriorates the performance of the scoring functions. Compared to Sippl’s PMF, the TIG formalism has been developed as a better quantification of Bayesian information [28]. Strikingly, we see here that TIG’s accuracy suffers more than PMF’s from the low information content of the backbone representation—thus producing the two worst accuracies of the entire benchmark, for the backbone and backbone beads (55.7% and 54.3%, respectively). Moreover, Cα, Cβ, and Cα + Cβ yielded better results with the TIG score, as it better incorporates the information of these atoms. However, this formalism seems incompatible with the amount of information carried by protein side-chains, as the side-chain-only and all-atom representations displayed poor performance. These conclusions were confirmed by the results of the rank prediction test (Table 3).

**Table 3:** Ranks predicted by using different structural representations, averaged for three categories of models. For the ‘‘near-native” and ‘‘good” models, the lower the value (*i.e.* the higher the rank), the more accurate the scoring function; for models of ‘‘poor” quality, however, the lower the rank, the better.

		Average predicted rank									
Model quality	Scoring	Cα	Cβ	Cα + Cβ	backbone	backbone + Cβ	side-chains	all-atom	BB	SC	BB + SC
Near-native	PMF	68.1	53.2	50.2	65.0	55.5	48.7	47.1	71.6	53.2	48.6
Good		123.4	131.3	125.6	123.9	123.8	126.4	124.6	139.6	128.2	126.2
Near-native	TIG	61.9	50.6	49.1	100.6	80.6	104.7	84.2	84.1	84.2	71.6
Good		121.9	125.9	124.1	128.2	126.2	141.0	131.9	125.5	130.6	125.6
Poor	PMF	222.9	220.9	230.5	224.4	229.8	230.5	234.9	203.6	225.4	231.3
Poor	TIG	229.3	230.0	234.2	202.8	215.3	186.3	207.0	212.1	207.8	220.1

**Fig. 2:**
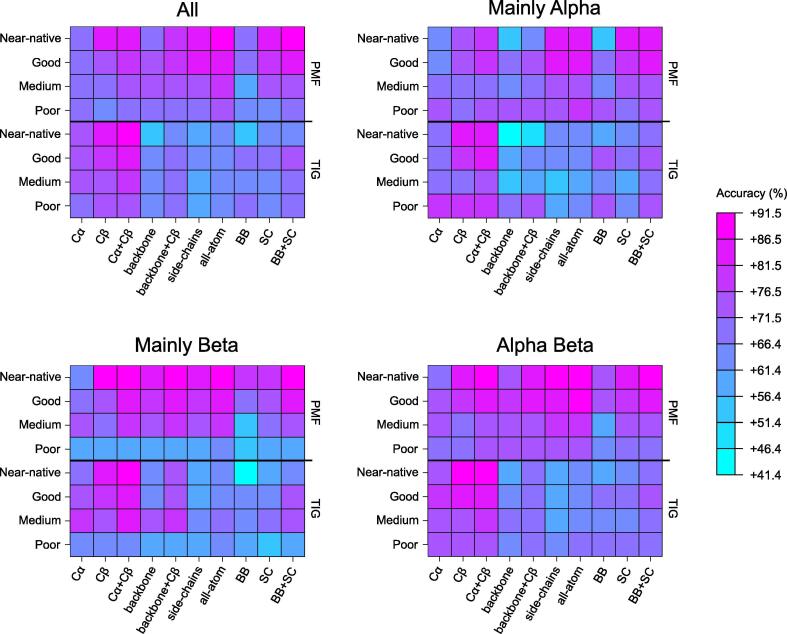
Color maps of the accuracies obtained for the different subsets of 3DRobot, as defined by he three main CATH classes. The exact values are provided in the Supplementary material.

To investigate whether the accuracy of each structural representation depends on the secondary structure content, we have divided the 200 proteins of 3DRobot into three subsets, based on CATH (version 4.3.0) [37] classes: “Mainly Alpha” (n = 60), “Mainly Beta” (n = 53), and “Alpha Beta” (n = 86)—the decoys of PDB 3a38A have been discarded, as this structure belongs to the “Few Secondary Structures” class. Thus, it appears that the structural class has some influence on the order by which the ten representations rank, for both PMF and TIG formalisms (Fig. 2). This is particularly true for the backbone, backbone + Cβ, and backbone beads representations, which perform poorly with the Mainly Alpha structures, while being substantially more accurate with the Mainly Beta ones. Given that the secondary structure elements are assigned based on backbone atoms, observing the most important differences on these backbone-based representations was not unexpected. The present results indicate that the backbones of Mainly Beta proteins contain considerably more information than those of Mainly Alpha ones. This may be explained by the fact that backbone atoms of adjacent beta strands often form hydrogen bonds which interconnect them into beta sheets, whereas interactions between alpha helices rather involve side-chain atoms. This higher information content makes easier the quality assessment of Mainly Beta models, except for those belonging to the “Poor” category. Interestingly, the Cβ representation also shows an increase in accuracy for Mainly Beta proteins, but to a lesser extent than the backbone-based ones. Although they are part of the side-chain, Cβ atoms are directly connected to the backbone and, therefore, may benefit from its aforementioned higher information content. Finally, it seems logical that the “Alpha Beta” subset shows only few differences with the whole 3DRobot set. From all these secondary structure-specific results, we can assume that dividing the training dataset based on the three main CATH classes could improve the accuracy of the scoring functions, although this requires further study.

Taken as a whole, these data led to the following top ranking: PMF_all-atom_, TIG_Cα+Cβ_, PMF_all-bead_, PMF_side-chains_, TIG_Cβ_, and PMF_Cα+Cβ_. Interestingly, the complexity of the second and third best representations, as measured by the number of atom pairs (Table 1), is one order of magnitude lower than that of PMF_all-atom_. Therefore, unlike PMF_side-chains_, they appear to be good compromises between speed and accuracy. The notable absence of TIG_all-atom_ from this top ranking—as its accuracy reached only 63.1% for the near-native models—may be a consequence of the redundancy (as opposed to information) content of the non-Cβ side-chain atoms, to which TIG is more sensitive, due to its very design. The TIG score performs best when using Cα + Cβ atoms, most likely because this representation optimally concentrates information about both backbone positioning and side-chain orientation. Additional use of any other atom type would then be redundant, thus degrading TIG performance. Finally, the relevance of the presented performances was shown by comparison with an external standard: the GOAP statistical potential [38], which relies on an all-atom representation of protein structures. In our previous work, it yielded accuracies of 91.5%, 86.8%, 80.8, and 76.2%, for the near-native, good, medium, and poor models, respectively. Its slight, yet significant, superiority over PMF_all-atom_ is presumably due to its double dependence on distances and angles, as for each heavy atom in interacting pairs, GOAP uses the relative orientation of the corresponding planes. For each of the five best scoring functions built here, the other validation was the measure of the correlation between the predicted quality and the TM-score, and its comparison with other methods, namely GOAP, SVMQA [21], OPUS-PSP [39], RWplus [40], and dDFIRE [41], [42] (Table 4). This last test showed that the results produced here for studying protein structure representations are comparable to the performance of current methods. Noteworthily, these data are consistent with the above accuracy ranking, except that PMF_all-atom_ equals GOAP.

**Table 4:** Correlation between the TM-score and the predicted quality, for different assessment programs. First column is the Pearson correlation coefficient (CC); second and third columns are the Spearman's (ρ) and Kendall's (τ) rank correlations coefficients, respectively. ^1^Values from [21].

Scoring	CC_rank_	ρ_rank_	τ_rank_
SVMQA^1^	0.910	0.882	0.713
OPUS-PSP^1^	0.807	0.752	0.570
GOAP^1^	0.883	0.849	0.671
RWplus^1^	0.834	0.806	0.624
DFIRE_GOAP_^1^	0.840	0.808	0.627
dDFIRE^1^	0.785	0.763	0.585
PMF_all-atom_	0.886	0.851	0.668
TIG_Cα+Cβ_	0.876	0.836	0.651
PMF_all-bead_	0.864	0.826	0.637
PMF_side-chains_	0.864	0.821	0.633
TIG_Cβ_	0.851	0.811	0.623
PMF_Cα+Cβ_	0.851	0.813	0.623

### 3.2 Speed benchmark

Besides accuracy, the other valuable criterion for selecting a scoring method is its speed, as the prediction of protein folding requires assessing thousands of conformations generated by the sampling algorithm. Here, we have measured the time taken by the five most accurate statistical potentials built for this work; GOAP was kept as an external standard method (Table 5). The results are no surprise, as the complexity of each representation can be estimated by the number of possible atom pairs (Table 1). Thus, PMF_all-atom_ is the slowest method, while TIG_Cβ_ is the fastest. For comparison purposes, we have included PMF_Cβ_ in the benchmark, as the time complexity of the PMF and TIG algorithms could be different. Indeed, depending on the processor architecture, the log function can be taken in either one or two assembly instructions, the latter case making the PMF algorithm more complex. However, both methods processed the 60,200 inputs at the same rate, while running on the same CPU. Finally, it is interesting to observe that the all-atom GOAP is ~2.5 faster than PMF_all-atom_, which could be due to a suboptimal C++ implementation of our algorithm.

**Table 5:** Time taken by the most accurate scoring functions for assessing 60,200 predicted structures. Each program ran on a single CPU.

Scoring	CPU time (minutes)
PMF_all-atom_	348
TIG_Cα+Cβ_	17
PMF_all-bead_	24
PMF_side-chains_	83
TIG_Cβ_	5
PMF_Cα+Cβ_	17
PMF_Cβ_	5
GOAP	140

### 3.3 Glycine and reference state representations

In the literature, distance-dependent statistical potentials are often misinterpreted as genuine interatomic potentials, with the idea that the inter-residue interaction profiles can find a direct physicochemical explanation. As a consequence, the fact that low-resolution statistical potentials are actually built from all the atom types found in the native structures may raise questions. If the observed frequency is derived from a Cα-only representation, why should the reference state be calculated using an all-atom one? In other respects, for a Cβ-only representation, is it valid to include glycine residues through their α carbons? Of note, the second question is relevant to the speed problem, as removing a residue would reduce the number of Cβ atom pairs from 210 to 171. The answers lie in the Bayesian view of these scoring functions, according to which (i) the reference frequency *P*(*d*_ij_|*M_2_*) of having two atoms of any type at a *d*_ij_ distance is called the *prior*, and (ii) the observed frequency *P*(*d*_ij_|*M*_1_) of having two atoms of specific residue types at a *d*_ij_ distance is called the *posterior*. Thus, statistical PMFs actually measure the amount of information incorporated into the background model *M_2_* for updating the prior to the posterior. Moreover, the log-likelihood ratio in Eq. (1) can be alternatively written as an information gain Δ*I*, where the definition of information is that of Shannon “surprisal”:

(3) $$\Delta I\left(d_{\mathrm{ij}}\right)=I_{\mathrm{prior}}-I_{\mathrm{posterior}}=-\text{log}\left(P\left(d_{\mathrm{ij}}|M_{\text{2}}\right)\right)-\left[-\text{log}\left(P\left(d_{\mathrm{ij}}|M_{\text{1}}\right)\right)\right]$$

For a posterior trained on α carbons, an all-atom-based prior will result in a greater Δ*I* than a Cα-based one. This is because the information added for the Bayesian updating will be that of the atom type, rather than that of the sole residue type; e.g. “UNK-xxxx → ALA-Cα”, rather than “UNK → ALA”, where UNK and xxxx are unknown residue and atom types, respectively. Therefore, this greater information gain shall result in a more accurate PMF scoring function. This also works for the TIG formalism, except with a generalized definition of information [28]. To support this theory, we built alternative Cβ PMF and TIG scoring functions, based on a reference state calculated from Cβ atoms only (Table 6).

**Table 6:** Accuracy in ranking models pairwise (n = 60,200) based on β carbons, depending on the exclusion of either other atoms in the reference state (‘Cβ_Cβ ref._’), or glycine residues (‘Cβ_no-Gly_’).

		Accuracy (%)		
Model quality	Scoring	Cβ_Cβ ref._	Cβ_no-Gly_	Cβ
Near-native	PMF	78.4	77.3	82.9
Good		76.6	77.0	76.1
Medium		72.1	72.3	67.8
Poor		62.2	62.7	66.4
Near-native	TIG	62.9	79.3	85.2
Good		68.3	78.9	80.8
Medium		66.4	78.5	74.0
Poor		60.7	69.3	71.6

With the PMF method, the disadvantage of using a Cβ-only reference state was confirmed for the near-native and poor models. However, accuracies were similar for the good category and, surprisingly, higher for the medium models. For the TIG method, the expected effect was dramatic for all types of models, with accuracy differences up to 22.3% for the near-native category. Similarly to what was observed in the accuracy benchmark section, the fact that TIG suffers more than PMF from the lower information gain of a Cβ-only reference state is due to its design based on information theory [28]. The Bayesian interpretation of the statistical potentials also explains why it is perfectly valid to include glycine Cα in a Cβ-only protein structure representation. We verified this by training Cβ-only PMF and TIG scoring functions that do not process the glycine residues. This led to lower accuracies for both PMF and TIG, except for the medium quality models. However, as shown by the TIG results, including glycine in a Cβ representation is not as important as training the reference state on all atom types.

### 3.4 Comparison with previous work

To this day, the choice of a protein structure representation was based either on results obtained by previously published methods, or on intuitions, such as that using all atoms is more accurate than lower resolutions, or that Cβ atoms carry more information than Cα ones. Indeed, for the past twenty years, the only comprehensive study researchers could rely on was the landmark article by F. L. Melo, R. Sánchez and A. Šali [43]. However, this work did not address the question of the speed and used a Z-score algorithm of higher time complexity than simple PMFs, thus restricting the possible representations to backbone atoms and β carbons. Moreover, limitations related to the then “knowledge” (*i.e.* data) of protein structures could now vindicate some of the conclusions drawn from these knowledge-based scoring functions. These limitations affect the training set, testing set, and metric: (i) the training set only contained 760 chains, although the sequence identity and X-ray resolution cutoffs were 30% and 2.5 Å, respectively; (ii) there was no large and balanced test set available, such as the benchmark-oriented 3DRobot; (iii) there was no TM-score nor GDT_TS, so that the assessment consisted of discriminating good and bad comparative models, which were labeled as such based on their template structures. Remarkably, despite these critical differences, we converged on the same conclusion that the combination of Cα and Cβ is the most accurate representation—among those tested by the authors—for a distance-dependent scoring function. Our study show that the then observed accuracies could have been further enhanced with the TIG score, or with side-chain atoms.

## 4 Conclusions and perspectives

In this work, we showed that, unsurprisingly, inter-residue interactions within protein structures are most accurately represented by using all heavy atoms. However, a better speed–accuracy trade-off was achieved with a Cα + Cβ representation and our recent TIG scoring method. Analogous to this is the MARTINI coarse-grained modeling, in which the protein backbone and side-chains are represented by BB and SC beads, rather than Cα and Cβ atoms, respectively. Although promising, it did not emerge here as the best option, as it ranked third in both accuracy and speed. Finally, the other best trade-off for protein structure prediction was that of the Cβ representation. Indeed, using only one atom for each residue optimizes speed, at the cost of a small decrease in accuracy, by comparison with high resolutions. Interestingly, this decrease was only observed for scoring functions built with the widely-used PMF method, as TIG showed best performances with the Cβ and Cα + Cβ representations. The sensitivity of the TIG method to the information and redundancy content of the input data further demonstrates its superiority over statistical PMFs. This is also supported by results obtained from training the reference state using only Cβ atoms.

The successful AlphaFold method also uses a Cβ representation. However, its statistical PMF combines interatomic distances and torsion angles. As the respective weights of these two types of structural features in the accuracy of AlphaFold’s scoring function remain unknown, future investigations will focus on processing dihedral angle values, as well as other descriptors, such as solvent accessibility or local conformations. We also showed that using α carbons of glycine residues was necessary for a Cβ representation. This raises the possibility of weighting the contribution of each residue type to the accuracy of the scoring function. Further efforts will concentrate on developing the MyPMFs tool in this direction. Finally, it should be brought to readers’ attention that knowledge-based scoring functions, now legitimized by AlphaFold, are applicable to RNA 3D structure [44]. Although the required experimental data might be currently too scarce for a deep learning approach, the simpler methods presented here are not specific to proteins and could be transposed to the RNA folding problem—which includes coarse-grained modeling, as the MARTINI force field has been recently extended to RNA macromolecules [45].

## CRediT authorship contribution statement

**Guillaume Postic:** Conceptualization, Formal analysis, Methodology, Software, Supervision, Validation, Writing - original draft, Writing - review & editing. **Nathalie Janel:** Funding acquisition, Writing - review & editing. **Gautier Moroy:** Formal analysis, Validation, Writing - original draft, Writing - review & editing.

## Declaration of Competing Interest

The authors declare that they have no known competing financial interests or personal relationships that could have appeared to influence the work reported in this paper.

## References

[1] Kennedy D., Norman C. (2005). What Don’t We Know?. Science.

[2] Zwanzig R., Szabo A., Bagchi B. (1992). Levinthal’s paradox. Proc. Natl. Acad. Sci..

[3] Rigden D.J. (2009). From protein structure to function with bioinformatics.

[4] Anfinsen C.B. (1973). Principles that Govern the Folding of Protein Chains. Science.

[5] Sieradzan A.K., Makowski M., Augustynowicz A., Liwo A. (2017). A general method for the derivation of the functional forms of the effective energy terms in coarse-grained energy functions of polymers. I. Backbone potentials of coarse-grained polypeptide chains. J. Chem. Phys..

[6] Liwo A., Khalili M., Czaplewski C., Kalinowski S., Ołdziej S., Wachucik K. (2007). Modification and Optimization of the United-Residue (UNRES) Potential Energy Function for Canonical Simulations. I. Temperature Dependence of the Effective Energy Function and Tests of the Optimization Method with Single Training Proteins. J. Phys. Chem. B.

[7] Miyazawa S., Jernigan R.L. (1985). Estimation of effective interresidue contact energies from protein crystal structures: quasi-chemical approximation. Macromolecules.

[8] Sippl M.J. (1990). Calculation of conformational ensembles from potentials of mena force: An approach to the knowledge-based prediction of local structures in globular proteins. J. Mol. Biol..

[9] Simons K.T., Kooperberg C., Huang E., Baker D. (1997). Assembly of protein tertiary structures from fragments with similar local sequences using simulated annealing and bayesian scoring functions11Edited by F. E. Cohen. J. Mol. Biol..

[10] Hamelryck T., Borg M., Paluszewski M., Paulsen J., Frellsen J., Andreetta C. (2010). Potentials of Mean Force for Protein Structure Prediction Vindicated, Formalized and Generalized. PLOS ONE.

[11] Valentin J.B., Andreetta C., Boomsma W., Bottaro S., Ferkinghoff-Borg J., Frellsen J. (2014). Formulation of probabilistic models of protein structure in atomic detail using the reference ratio method. Proteins Struct. Funct. Bioinforma.

[12] Hamelryck T., Boomsma W., Ferkinghoff-Borg J., Foldager J., Frellsen J., Haslett J. (2015). Proteins, physics and probability kinematics: a Bayesian formulation of the protein folding problem. Geom. Driven Stat., John Wiley & Sons, Ltd.

[13] Zhao F., Peng J., Xu J. (2010). Fragment-free approach to protein folding using conditional neural fields. Bioinformatics.

[14] Li J., Fu A., Zhang L. (2019). An Overview of Scoring Functions Used for Protein-Ligand Interactions in Molecular Docking. Interdiscip. Sci. Comput. Life Sci..

[15] Pucci F., Kwasigroch J.M., Rooman M., Gáspári Z. (2020). Protein Thermal Stability Engineering Using HoTMuSiC. Struct. Bioinforma. Methods Protoc..

[16] Pucci F., Kwasigroch J.M., Rooman M. (2017). SCooP: an accurate and fast predictor of protein stability curves as a function of temperature. Bioinformatics.

[17] Hou Q., Kwasigroch J.M., Rooman M., Pucci F. (2020). SOLart: a structure-based method to predict protein solubility and aggregation. Bioinformatics.

[18] Orlando G., Silva A., Macedo-Ribeiro S., Raimondi D., Vranken W. (2020). Accurate prediction of protein beta-aggregation with generalized statistical potentials. Bioinformatics.

[19] Alford R.F., Leaver-Fay A., Jeliazkov J.R., O’Meara M.J., DiMaio F.P., Park H. (2017). The Rosetta All-Atom Energy Function for Macromolecular Modeling and Design. J. Chem. Theory Comput..

[20] Uziela K., Wallner B. (2016). ProQ2: estimation of model accuracy implemented in Rosetta. Bioinformatics.

[21] Manavalan B., Lee J. (2017). SVMQA: support–vector-machine-based protein single-model quality assessment. Bioinformatics.

[22] Zhao F., Xu J. (2012). A Position-Specific Distance-Dependent Statistical Potential for Protein Structure and Functional Study. Structure.

[23] Senior A.W., Evans R., Jumper J., Kirkpatrick J., Sifre L., Green T. (2020). Improved protein structure prediction using potentials from deep learning. Nature.

[24] Nugent T., Jones D.T. (2013). Membrane protein orientation and refinement using a knowledge-based statistical potential. BMC Bioinf..

[25] Marrink S.J., Risselada H.J., Yefimov S., Tieleman D.P., de Vries A.H. (2007). The MARTINI Force Field: Coarse Grained Model for Biomolecular Simulations. J. Phys. Chem. B.

[26] Bruininks B.M.H., Souza P.C.T., Marrink S.J., Bonomi M., Camilloni C. (2019). A Practical View of the Martini Force Field. Biomol. Simul. Methods Protoc..

[27] Scott D.W. (2015). Multivariate Density Estimation: Theory, Practice, and Visualization.

[28] Postic G., Janel N., Tufféry P., Moroy G. (2020). An information gain-based approach for evaluating protein structure models. Comput. Struct. Biotechnol. J..

[29] Postic G., Hamelryck T., Chomilier J., Stratmann D. (2018). MyPMFs: a simple tool for creating statistical potentials to assess protein structural models. Biochimie.

[30] Wang G., Dunbrack R.L. (2003). PISCES: a protein sequence culling server. Bioinformatics.

[31] Wang G., Dunbrack R.L. (2005). PISCES: recent improvements to a PDB sequence culling server. Nucleic Acids Res..

[32] Deng H., Jia Y., Zhang Y. (2016). 3DRobot: automated generation of diverse and well-packed protein structure decoys. Bioinformatics.

[33] Zhang Y., Skolnick J. (2004). Scoring function for automated assessment of protein structure template quality. Proteins Struct. Funct. Bioinforma.

[34] de Jong D.H., Singh G., Bennett W.F.D., Arnarez C., Wassenaar T.A., Schäfer L.V. (2013). Improved Parameters for the Martini Coarse-Grained Protein Force Field. J. Chem. Theory Comput..

[35] Joosten R.P., Joosten K., Murshudov G.N., Perrakis A. (2012). PDB_REDO: constructive validation, more than just looking for errors. Acta Crystallogr. D Biol. Crystallogr..

[36] Joosten R.P., Long F., Murshudov G.N., Perrakis A. (2014). The PDB_REDO server for macromolecular structure model optimization. IUCrJ.

[37] Sillitoe I., Bordin N., Dawson N., Waman V.P., Ashford P., Scholes H.M. (2021). CATH: increased structural coverage of functional space. Nucleic Acids Res..

[38] Zhou H., Skolnick J. (2011). GOAP: A Generalized Orientation-Dependent, All-Atom Statistical Potential for Protein Structure Prediction. Biophys. J..

[39] Lu M., Dousis A.D., Ma J. (2008). OPUS-PSP: An Orientation-dependent Statistical All-atom Potential Derived from Side-chain Packing. J. Mol. Biol..

[40] Zhang J., Zhang Y. (2010). A Novel Side-Chain Orientation Dependent Potential Derived from Random-Walk Reference State for Protein Fold Selection and Structure Prediction. PLoS ONE.

[41] Yang Y., Zhou Y. (2008). Specific interactions for ab initio folding of protein terminal regions with secondary structures. Proteins Struct. Funct. Bioinforma.

[42] Yang Y., Zhou Y. (2008). Ab initio folding of terminal segments with secondary structures reveals the fine difference between two closely related all-atom statistical energy functions. Protein Sci..

[43] Melo F., Sánchez R., Sali A. (2002). Statistical potentials for fold assessment. Protein Sci..

[44] Tan Y.-L., Feng C.-J., Jin L., Shi Y.-Z., Zhang W., Tan Z.-J. (2019). What is the best reference state for building statistical potentials in RNA 3D structure evaluation?. RNA.

[45] Uusitalo J.J., Ingólfsson H.I., Marrink S.J., Faustino I. (2017). Martini Coarse-Grained Force Field: Extension to RNA. Biophys. J..

