# Supplementary material for: Representations of protein structure for exploring the conformational space: A speed–accuracy trade-off
Source: Comput Struct Biotechnol J. 2021 Apr 28;19:2618–25. doi: 10.1016/j.csbj.2021.04.049 (PMC8120936; doi:10.1016/j.csbj.2021.04.049)
Supplement: Supplementary data 1 [file mmc1.doc]

**SUPPLEMENTARY MATERIAL**

**Representations of protein structure for exploring the conformational space: a speed–accuracy trade-off**

Guillaume Postic^1^*, Nathalie Janel^2^, Gautier Moroy^1^

^1^Université de Paris, BFA, UMR 8251, CNRS, ERL U1133, Inserm, F-75013 Paris, France

^2^Université de Paris, BFA, UMR 8251, CNRS, F-75013 Paris, France

*To whom correspondence should be addressed: [guillaume.postic@u-paris.fr](mailto:guillaume.postic@u-paris.fr)

**Table S1**. Accuracy in ranking models pairwise for the “Mainly Alpha” CATH class.

**Table S2**. Accuracy in ranking models pairwise for the “Mainly Beta” CATH class.

**Table S3**. Accuracy in ranking models pairwise for the “Alpha Beta” CATH class.
